# Assessing Lung Involvement in Paediatric Rheumatic Diseases: A Retrospective Evaluation of the Limitations of Spirometry

**DOI:** 10.31138/mjr.020325.pae

**Published:** 2025-08-18

**Authors:** Spyridon Prountzos, Kyveli Chiotopoulou, Katerina Kourtesi, Evdoxia Sapountzi, Dafni Moriki, Elpiniki Kartsiouni, Euthymia Alexopoulou, Konstantinos Douros, Lampros Fotis

**Affiliations:** 1 2^nd^ Department of Radiology, Attikon General Hospital, National and Kapodistrian University of Athens, Greece;; 2 Division of Paediatric Rheumatology, 3^rd^ Department of Paediatrics, Attikon General Hospital, National and Kapodistrian University of Athens, Greece;; 3 2^nd^ Department of Paediatrics, AHEPA General Hospital, Aristotle University of Thessaloniki, Greece;; 4 Division of Paediatric Pulmonology and Allergology, 3^rd^ Department of Paediatrics, Attikon General Hospital, National and Kapodistrian University of Athens, Greece

**Keywords:** lung, lung diseases, interstitial, pediatrics, rheumatic diseases, spirometry, tomography, x-ray computed

## Abstract

**Objective:**

Lung involvement in paediatric rheumatic diseases significantly impacts morbidity and mortality. High-resolution computed tomography (HRCT) is a sensitive diagnostic tool but raises concerns about radiation exposure. Spirometry, a non-invasive and accessible test, is often used to assess pulmonary function, but its accuracy in detecting lung disease in newly diagnosed pediatric rheumatic conditions remains uncertain. This study evaluates spirometry’s reliability, using HRCT as the reference standard.

**Methods:**

Patients suspected of lung involvement due to respiratory symptoms or disease prevalence underwent HRCT and pulmonary assessment. A retrospective review of HRCT scans was conducted for 22 pediatric patients diagnosed with rheumatic disease (January 2021–December 2023), all of whom had pathological findings. HRCT was performed using a paired end-inspiratory and forced-expiratory protocol with 1-mm collimation. Radiological findings, including parenchymal opacities, ground-glass opacities, reticular patterns, honeycombing, parenchymal bands, bronchiectasis, peribronchial wall thickening, and air trapping, were assessed. Spirometric values—percent predicted Forced Expiratory Volume in 1 second (ppFEV1), Forced Vital Capacity (ppFVC), and FEV1/FVC ratio—were collected for patients with acceptable flow-volume curves.

**Results:**

Seventeen of 22 patients (77.3%) had technically acceptable spirometry. Compared to HRCT, spirometry demonstrated a sensitivity of 29.4%.

**Conclusion:**

Despite its excellent positive predictive value, spirometry’s low sensitivity suggests it may miss early lung involvement in paediatric rheumatic diseases. HRCT remains the preferred diagnostic tool to ensure accurate detection and management, despite radiation concerns.

## INTRODUCTION

Rheumatic diseases are a group of autoimmune disorders that affect multiple organs, with the lungs being a common target. Individual or several components of the respiratory system, including the airways, vessels, parenchyma, pleura, and respiratory muscles, may be related to the illness itself or to the medications used. Lung involvement is a major determinant of patient morbidity and mortality, while the pattern of lung disease is considerably heterogeneous in incidence, prevalence, and severity depending on the underlying rheumatic disease.^1,2^

Systemic inflammatory diseases with the highest likelihood of pulmonary involvement are juvenile systemic lupus erythematosus (SLE), scleroderma (systemic sclerosis [SSc]), juvenile dermatomyositis (JDM), mixed connective tissue disease (MCTD), granulomatosis with polyangiitis and systemic juvenile idiopathic arthritis (JIA)/Still’s disease.^3^ Interstitial lung disease (ILD) is usually the most frequent presentation of lung involvement in adult patients, while bronchiectasis, and obstructive airways disease may also occur. The incidence of pulmonary complications is lower in childhood, and some forms of diffuse lung disease are unique to infants and children.^4^ Clinically significant pulmonary complications are relatively uncommon in childhood, and, if present, may require changes in disease management.^5^

Recognition of pulmonary involvement depends on the methods used to detect the disease. Although various tests are available for assessing lung function, their sensitivity and specificity in detecting lung involvement in newly diagnosed patients are not always consistent with their efficacy in monitoring lung function throughout the course of inflammatory rheumatic disease.^6^

Children’s Interstitial Lung Disease (chILD) encompasses a diverse group of rare, chronic lung disorders affecting children and infants. These diseases are marked by a range of symptoms, such as persistent cough, shortness of breath, hypoxaemia, and reduced exercise tolerance, which reflect underlying diffuse lung pathology.^7^ Diagnostic imaging, particularly high-resolution computed tomography (HRCT), is crucial in chILD, revealing characteristic patterns like hyperinflation, mosaic attenuation, air trapping, ground-glass opacities, consolidation, linear or reticular opacities, nodules, and cysts.^8^

HRCT is the most effective tool for detecting lung disease, as it is highly sensitive in identifying morphological abnormalities.^9^ However, concerns about radiation exposure, particularly in children, necessitate careful use. Other non-invasive and radiation-free tests, such as lung ultrasound and spirometry, have been proposed as alternatives.^6,10^ Spirometry in particular is a simpler, non-invasive method for assessing pulmonary function and is widely used for screening and monitoring lung function and involvement in patients with inflammatory rheumatic diseases.^6^ A multidisciplinary group of physicians who developed a screening algorithm for lung disease in patients with JIA, proposed the use of pulmonary function tests (PFTs), including spirometry, as the first-line assessment for patients with at least one of nine red flag features, followed by HRCT in those with abnormal results.^11^ Despite its accessibility, the reliability of spirometry in detecting lung disease in children with newly diagnosed rheumatic conditions is uncertain.^6^

In this study, we aimed to evaluate the diagnostic accuracy of spirometry in detecting lung involvement in a population of newly diagnosed children with rheumatic diseases, using HRCT as the gold standard.

## METHODS

The study was conducted between January 2021 and December 2023. Demographic data, clinical characteristics, spirometry results and HRCT imaging findings were retrospectively collected. In our institution, all patients suspected of having lung involvement, either due to the presence of respiratory-related symptoms or the high prevalence of respiratory involvement associated with the specific disease^12^_,_ undergo a high-resolution computed tomography (HRCT) scan. The patients were treatment-naïve and had been diagnosed with rheumatic disease for the first time. Patients underwent chest CT examination using paired end-inspiratory and forced-expiratory scan protocol using 1-mm collimation and slice thickness reconstruction algorithm. Only patients with radiological findings were included in the study. Parenchymal opacities, ground-glass opacities (GGOs), reticular pattern, honeycombing, parenchymal bands, bronchiectasis, and peribronchial wall thickening were examined. Air trapping was assessed during expiratory scans. Children with a significant combination of hyperinflation, mosaic attenuation, air trapping, ground-glass opacities, consolidation, linear or reticular opacities, nodules, or cysts were defined as having lung disease consistent with chILD.

Spirometry was performed in all patients, and the percent predicted values for Forced Expiratory Volume in 1 second (ppFEV1) and Forced Vital Capacity (ppFVC), along with the FEV1/FVC ratio, were recorded. Only patients with technically acceptable flow-volume curves were included in the analysis. Patients with reduced ppFEV1 and/or a reduced FEV1/FVC ratio were classified as having obstructive lung disease, while those with reduced ppFVC and a normal FEV1/FVC ratio were classified as having restrictive lung disease.

All symptoms associated with the respiratory tract were reviewed. Exclusion criteria were history of chronic lung or cardiac diseases, asthma, cystic fibrosis, immunodeficiency, congenital heart disease, bronchopulmonary dysplasia, pulmonary infection and recurrent aspiration.

All patients fulfilled the commonly accepted classification criteria for their respective diagnoses. For SLE, the 2019 European League Against Rheumatism (EULAR)/American College of Rheumatology (ACR) classification criteria were used.^13^ Systemic sclerosis (SSc) was diagnosed based on the Paediatric Rheumatology European Society (PRES)/ACR/EULAR provisional classification criteria.^14^ Juvenile dermatomyositis (JDM) was classified according to the Peter and Bohan diagnostic criteria,^15^ and systemic juvenile idiopathic arthritis (JIA) was diagnosed using the 2019 Paediatric Rheumatology International Trials Organisation (PRINTO) criteria.^16^ Mixed connective tissue disease (MCTD) was diagnosed based on Kasukawa’s criteria,^17^ while granulomatosis with polyangiitis (GPA) was classified using the EULAR/PRINTO/PRES 2008 Ankara criteria.^18^

For Behçet’s disease, the paediatric Behçet’s disease criteria were applied.^19^ No formal classification criteria exist for microscopic polyangiitis, and the patient with unclassified vasculitis did not meet any of the available vasculitis criteria. Similarly, the patient with pulmonary hemosiderosis was diagnosed by exclusion, as no specific classification criteria exist for this condition.

### Statistical analysis

Our study focused exclusively on patients with definitive radiological findings. However, the absence of variability in the outcome variable hindered our ability to conduct a receiver operating characteristic analysis or derive a comprehensive set of accuracy metrics. As a result, we could only calculate sensitivity based on the available data on false negatives.

The study protocol was approved by the Ethics Committee of Attikon University Hospital, National and Kapodistrian University of Athens, Athens, Greece. Patient confidentiality was maintained throughout the study in accordance with ethical guidelines.

## RESULTS

Twenty-two paediatric patients diagnosed with various rheumatic diseases that exhibited one or more pathologic findings in their HRCT were included in the study. The demographic and clinical characteristics, the specific diagnoses and the radiological HRCT findings are detailed in **Table 1**. Twelve of the 22 patients (54.5%) had HRCT patterns consistent with chILD. Only 17 (77.3%) patients provided a technically acceptable spirometric flow-volume curve. Mean (sd) values for ppFVC, ppFEV1, and FEV1/FVC ratio were 90.71 (13.33), 95.06 (17.54), and 91.48 (8.63), respectively. Four (18.2%) patients had spirometric values suggestive of restrictive and one (4.5%) of obstructive lung disease. None of the first-degree relatives of the study participants had a prior diagnosis of ILD.

**Table 1.** Demographic, clinical, and radiological characteristics of enrolled patients.

**Variable**	**Categories**	**N (%)**
Sex	Female	18 (81.8%)
	Male	4 (18.2%)

Age (in years)	Mean 13.05 (±3.169, min 3, max 16)	

Cough	Yes	6 (27.3%)
	No	16 (72.7%)

Dyspnoea	Yes	3 (13.6%)
	No	19 (87.4%)

Exercise intolerance	Yes	9 (40.9%)
	No	13 (59.1%)

Disease	Systemic lupus erythematosus	4 (18.2%)
	Systemic sclerosis	4 (18.2%)
	Juvenile dermatomyositis	3 (13.6%)
	Systemic Juvenile Idiopathic Arthritis	3 (13.6%)
	Mixed connective tissue disease	3(13.6%)
	Microscopic polyangiitis	1(4.5%)
	Granulomatosis with polyangiitis	1(4.5%)
	Non-specific vasculitis	1(4.5%)
	Behçet’s disease	1(4.5%)
	Idiopathic Pulmonary Hemosiderosis	1(4.5%)
HRCT scan	Yes	22 (100%)
	No	0 (0%)
Parenchymal opacities	Yes	7 (31.8%)
	No	15 (68.2%)
Ground-glass opacities	Yes	13 (59.1%)
	No	9 (40.9%)
Reticular pattern	Yes	10 (45.5%)
	No	12 (54.5%)
Honeycombing	Yes	1 (4.5%)
	No	21 (95.5%)
Parenchymal bands	Yes	12 (54.5%)
	No	10 (45.5%)
Bronchiectatic changes	Yes	9 (40.9%)
	No	13 (59.1%)
Peribronchial wall thickening	Yes	19 (86.4%)
	No	3 (13.6%)
Mosaic attenuation	Yes	11 (50.0%)
	No	11 (50.0%)

Using HRCT scan findings as the reference standard the spirometry had a sensitivity of 29.4%.

## DISCUSSION

Lung involvement is a recognised complication of systemic rheumatic diseases, although it is less common in paediatric patients compared to adults.^2^ Spirometry is commonly employed in clinical practice for assessing lung function, given its simplicity and non-invasive nature. However, its diagnostic accuracy in pulmonary involvement in children with rheumatic diseases have remained a subject of debate. The findings of the present study highlight the limitations of spirometry in detecting lung involvement in children with newly diagnosed rheumatic diseases as it demonstrated poor sensitivity compared to HRCT, which identified abnormalities even in asymptomatic patients. This suggests that spirometry alone may not be sufficient for detecting early or subtle lung pathology, potentially leading to underdiagnosis and delays in appropriate management, especially in asymptomatic patients. Given the ability of HRCT to identify lung involvement regardless of the presence of respiratory symptoms, a more comprehensive approach incorporating imaging may be necessary for accurate assessment in this population. This aligns with previous reports highlighting spirometry’s limitations in early detection of pulmonary abnormalities compared to HRCT.^20,21^ ILD, ground-glass opacities (GGO), and bronchiectasis were frequently observed in HRCT, consistent with previous studies that identified these as common features of lung involvement in rheumatic diseases.^20^

Earlier studies have reported varying results regarding spirometry’s effectiveness in paediatric cohorts. In the study by Huang et al, 56.3% of children with newly diagnosed rheumatic disease had abnormal pulmonary function tests (PFTs), while only 16.7% showed abnormal HRCT findings. In the same study, half of the patients with abnormal HRCT results had normal PFTs.^21^ Similarly, Lilleby et al. found that PFTs were abnormal in 37% of their patients with childhood-onset SLE, whereas only 8% had abnormal HRCT findings.^22^ Ring et al. reviewed 11 studies on pulmonary function tests (PFTs) in children with rheumatic diseases. Abnormal spirometry was common, especially in SLE (100%) and in up to 50–60% of JDM and SSc cases, often in asymptomatic patients. However, one JDM study found normal spirometry in 82% of patients.^23^ Céspedes-Cruz et al. found ILD in 10 of 15 juvenile SSc patients, though only 2 had abnormal spirometry.^24^ In contrast, Doğantan et al. found mostly normal spirometry in JIA patients, with normal high-resolution CT (HRCT) findings.^25^ Consistently with our results, Veiga et al. showed that, in their cohort of patients with childhood-onset SLE, 70% had abnormal CT findings, which were minimal in 43%.^26^ This study, along with our data, is aligned with studies in adult patients that have reported a high prevalence of chest CT abnormalities suggestive of lung involvement and ILD mostly in asymptomatic patients with rheumatic diseases who have normal PFTs.^27^

A prior study evaluating a stepwise diagnostic screening approach, combining PFTs, chest radiography, and pulmonary HRCT, for detecting ILD in adult patients with immune-related diseases showed that HRCT had the highest sensitivity (100%) but lower specificity compared to PFTs and chest X-ray.^28^ Like the current paediatric study, GGOs was the most common HRCT finding among these adult patients. The combination of PFTs such as diffusing capacity of the lung for carbon monoxide (DLCO) <80% and chest X-ray increased sensitivity and specificity, suggesting that patients with reduced DLCO or suspicious chest X-ray findings should undergo HRCT to confirm ILD and exclude other pulmonary conditions.^28^ This suggests that HRCT despite involving radiation exposure, offers an accurate diagnostic tool for detecting ILD and other pulmonary abnormalities.

When assessing paediatric rheumatic patients, the choice between HRCT and PFTs should be carefully considered. HRCT’s sensitivity for detecting early or asymptomatic structural changes contrasts with the safer, more easily repeatable PFTs, which may be challenging for younger children to perform reliably.^29^ Similar issues may arise with HRCT, although sedation can be administered when necessary to facilitate the process. While HRCT is more sensitive, its use in children is limited due to radiation exposure risks and the subclinical nature of lung disease in asymptomatic patients.^6^ Nevertheless, chest CT scan is considered the gold standard method for defining structural abnormalities.^30^ Recent advances in CT technology have enabled faster scan times with super low-dose options, achieving dose-length (DLPs) as low as 7.66 mGy*cm per scan.^31^ However, in paediatric populations, the concern about long-term radiation effects remains, especially for repeated scans. Despite these concerns, HRCT is still recommended for screening in high-risk paediatric patients.^21^.

Detecting ILD in paediatric rheumatic patients presents challenges distinct from those in adults. In adults, HRCT and PFTs are widely recognised as crucial tools for screening and monitoring ILD, with HRCT being highly sensitive in detecting early structural abnormalities, even before functional impairments manifest, as indicated by reduced pulmonary function parameters like FVC and DLCO.^32^ The guidelines published by the American College of Rheumatology and the American College of Chest Physicians for people with systemic autoimmune rheumatic disease at increased risk of developing ILD, conditionally recommended HRCT and PFTs over PFTs alone for systemic autoimmune rheumatic diseases at high risk for ILD.^32^

While genetic factors have been implicated in ILD, none of the first-degree relatives in our study population had a prior diagnosis of ILD. This suggests that familial clustering may not be a prominent feature in this cohort. It is possible that environmental exposures or disease-specific factors play a more significant role in ILD development in this population. Further studies incorporating genetic analysis may help clarify the contribution of inherited predisposition.^33^

Certain limitations of this study include a small sample size which limits generalisability and the inclusion of various rheumatic diseases, each with distinct rates of lung involvement. Also, patients were not subjected to additional PFTs such as DLCO or plethysmography, which could have provided more comprehensive assessments of lung function. Future studies should include larger, more homogeneous cohorts, and a broader range of diagnostic tests to better understand PFTs sensitivity and specificity in comparison with spirometry or HRCT. Such research could further refine the balance between HRCT’s diagnostic sensitivity and its safe application in younger populations.

In conclusion, this study adds new insights to previously published articles in the Mediterranean Journal of Rheumatology by focusing on early lung involvement in paediatric rheumatic diseases, an area largely unexplored in prior research, which has primarily examined ILD in adults. Unlike previous studies that rely on imaging and serological markers, we specifically assess the limitations of spirometry in detecting early lung disease at the time of diagnosis. In addition, HRCT has a critical role in identifying subclinical pulmonary involvement in paediatric patients newly diagnosed with rheumatic diseases, findings not always apparent through symptoms or spirometry alone. Given the heterogeneous nature of lung disease in these conditions and the associated risks, this study supports HRCT’s inclusion in initial evaluations. Further research to refine diagnostic and monitoring strategies, could be pivotal in enhancing long-term respiratory outcomes for this vulnerable population.

## AUTHOR CONTRIBUTIONS

Conceptualisation, SP, EA, KD and LF.; methodology, SP, KD, LF.; formal analysis, KD; writing—original draft preparation, SP, KC, ES, KK, DM, EK; writing—review and editing, EA, KD, LF; supervision, KC, KK, LF. All co-authors take full responsibility for the integrity and accuracy of all aspects of the work.

## FUNDING

No specific funding was received from any bodies in the public, commercial or not-for-profit sectors to carry out the work described in this article.

## INSTITUTIONAL REVIEW BOARD STATEMENT

The study was conducted in accordance with the Declaration of Helsinki and approved by the Ethics Committee of Attikon General Hospital, NKUA EBD494, date 02/07/2024.

## INFORMED CONSENT STATEMENT

Patient consent was not mandated according to current practice for retrospective studies in our institution when only a secondary review of imaging data and retrospective clinical data is undertaken.

## DATA AVAILABILITY STATEMENT

The clinical data supporting the findings of this study are available from the corresponding author upon reasonable requests via email at lafotis@med.uoa.gr.

## CONFLICTS OF INTEREST

The authors declare no relevant conflicts of interest.

## DISCLAIMER

No part of this manuscript, including the text and graphics, are copied or published elsewhere in whole or in part.
